# Otorhinolaryngological Manifestations and Esophageal Disorders in Celiac Disease: A Narrative Review

**DOI:** 10.3390/jcm12227036

**Published:** 2023-11-10

**Authors:** Herbert Wieser, Carolina Ciacci, Carolina Gizzi, Antonella Santonicola

**Affiliations:** 1 Hamburg School of Food Science, Institute of Food Chemistry, University of Hamburg, 20146 Hamburg, Germany; h.wieser2@gmx.de; 2 Gastrointestinal Unit, Department of Medicine, Surgery and Dentistry “Scuola Medica Salernitana”, University of Salerno, 84131 Salerno, Italy; carolinagizzi@libero.it (C.G.); antonellasantonicola83@gmail.com (A.S.)

**Keywords:** aphthous ulcers, celiac disease, eosinophilic esophagitis, gluten-free diet, hearing loss, recurrent aphthous stomatitis, sleep apnea, xerostomia

## Abstract

Celiac disease (CeD) is a chronic gluten-sensitive immune-mediated enteropathy characterized by numerous intestinal and extra-intestinal signs and symptoms. Among extra-intestinal manifestations, otorhinolaryngological (ORL) complaints in CeD are relatively rare and their relation to CeD is frequently overlooked by physicians. Recent studies underlined that the prevalence of recurrent aphthous stomatitis, aphthous ulcers, geographic tongue, and xerostomia was significantly increased in CeD patients compared with healthy individuals. However, data about the other oral manifestations of CeD, such as atrophic glossitis, glossodynia, angular cheilitis, and salivary abnormalities, are scanty. Further ORL conditions associated with CeD include sensorineural hearing loss, nasal abnormalities, and obstructive sleep apnea. Moreover, several esophageal disorders such as gastroesophageal reflux disease and eosinophilic esophagitis have been associated with CeD. The pathophysiological link between both ORL and esophageal manifestations and CeD might be further investigated. In addition, also the role of gluten-free diet in improving these conditions is largely unclear. Certainly, otorhinolaryngologists can play an important role in identifying people with unrecognized CeD and may help prevent its long-term complications. The aim of this narrative review is to analyze the latest evidence on the association between CeD and ORL and esophageal manifestations.

## 1. Introduction

Celiac disease (CeD) is an immune-mediated enteropathy in genetically predisposed individuals caused by the ingestion of gluten present in wheat, rye, barley, and oat products. CeD is one of the most common food hypersensitivities worldwide. Recent epidemiological data suggest that the prevalence of diagnosed CeD is approximately 1% of the global population [1]. However, a considerable portion of people suffer from undetected CeD. A meta-analysis, comprising data from 291,969 study participants, revealed that the pooled prevalence of undetected CeD in female participants was 0.59% and in male participants was 0.42% [2]. Moreover, considering that the diagnosis of CeD is complex and often difficult, delays in diagnosis are common. For example, 54% of 611 adult Finnish CeD patients reported a diagnostic delay of more than three years [3]. A strict and lifelong gluten-free diet (GFD) is currently the only effective treatment strategy and usually results in prompt relief of clinical symptoms.

CeD manifestations include a variety of intestinal and extra-intestinal symptoms and can affect almost every organ of the body. Apart from classical gastrointestinal presentations such as chronic diarrhea, abdominal pain, bloating, and vomiting, many extra-intestinal symptoms such as nutritional deficiencies, reproductive problems, and neurological and psychiatric disorders may appear [4]. In some cases, extra-intestinal symptoms are the only clinical manifestations of CeD that frequently remain undiagnosed. The oral cavity is likewise one of the areas of extra-intestinal signs and symptoms [5,6,7]. For example, dental complaints, particularly dental enamel defects, are well-known manifestations of CeD [8]. Less known are the relations between CeD and oral soft tissue lesions. Additionally, there is still a lack of knowledge regarding hearing loss, nasal abnormalities, and sleep apnea in CeD. Patients with these complaints may be presented to otorhinolaryngologists initially and, therefore, these medical specialists should be aware of the possibility of concomitant CeD in order to prevent the progression and the long-term complications of unrecognized CeD.

The aim of this narrative review is to analyze the latest evidence on the association between CeD and otorhinolaryngological (ORL) and esophageal manifestations.

## 2. Materials and Methods

**Figure 1:**
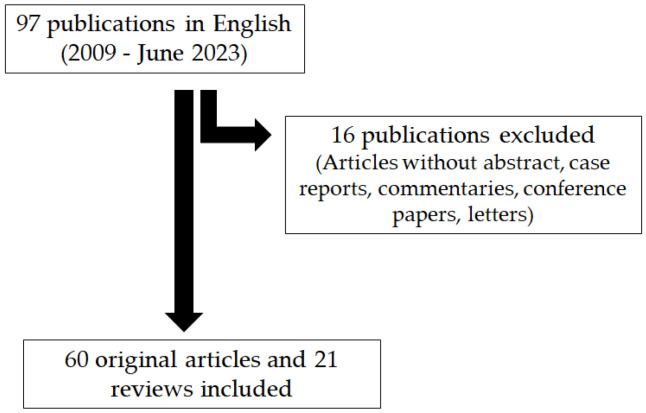
PRISMA flow diagram of search procedure.

PubMed database searches were performed for articles published in English from 2009 to June 2023. The searched keywords were “c(o)eliac disease“ in combination with ”oral manifestations“, “oral health”, “aphthous stomatitis”, “aphthous ulcers”, “atrophic glossitis”, “xerostomia”, “eosinophilic esophagitis”, “hearing loss”, “epistaxis”, and “sleep apnea”. We retrieved a total of 97 publications, of which 21 were reviews. We evaluated 60 original studies on the topic and reported all of them. Additional papers were selected from personal files on CeD and by cross-referencing from the retrieved articles. Exclusion criteria were only based on the type of publication. Articles without abstracts, such as case reports, commentaries, conference papers, and letters, were excluded (Figure 1).

## 3. Oral Soft Tissue Manifestations

It is well known that a considerable number of gastrointestinal disorders can affect the oral cavity. Among these, for example, there are gastroesophageal reflux disease (GERD), ulcerative colitis, Crohn’s disease, and CeD [9]. Numerous studies both on children and adults have investigated the manifestations of the soft oral tissue associated with CeD. Van Gils et al. [10] reported one of the most extensive examinations of oral health problems of CeD patients in comparison to healthy individuals. A cohort of 740 Dutch patients with CeD (median age 55 years) and 270 controls (median age 53 years) were evaluated for the presence of oral manifestations by an online questionnaire. The first section of the inquiry recorded the presence of problems during the last 12 months and the second section consisted of the Oral Health Impact Profile 14 (OHIP-14). Twelve complaints (aphthous stomatitis, bad breath, temporomandibular joint problems, angular cheilitis, bad taste, painful mouth, diminished taste, inflammation oral mucosa, problems with eating and drinking, discoloration of the oral mucosa, glossodynia, difficulty speaking) were registered. The prevalence of all the oral health problems collected was significantly higher in CeD patients compared with controls. The authors stated that oral health complaints in patients diagnosed with CeD seem to be an underestimated problem in clinical practice. They suggested that otorhinolaryngologists should be aware of the possibility of CeD in patients with oral abnormalities, especially when other potential CeD-related symptoms are present, and they should consider referring these patients to a general practitioner or gastroenterologist for further evaluation [10].

Bramanti et al. [11] investigated the frequency of five specific oral soft tissue complaints (recurrent aphthous stomatitis, geographic tongue, burning tongue, atrophic glossitis, and angular cheilitis) in 50 children with ascertained CeD (mean age 7.5 years), 21 subjects with potential CeD (mean age 6.9 years), and 54 healthy controls (mean age 8.8 years). The overall prevalence of these tissue lesions was 62% in ascertained CeD, 76% in potential CeD, and 13% in controls. The difference between CeD patients and controls was highly significant (*p* < 0.05). The authors concluded that the preventive recognition of these specific oral lesions by the dentist or otorhinolaryngologist should allow preventing the disease’s manifestations on the mucosa. Moreover, patient health can be directed to better prognosis thanks to a suspected diagnosis of CD, avoiding and anticipating the occurrence of gastrointestinal symptoms and more severe pathological injury.

A recent systematic review by Lucchese et al. [7] aimed to catalog and characterize oral manifestations in CeD. Apart from dental enamel defects and delayed dental eruption, articles on complaints of the soft oral tissue were summarized. The information extracted from the studies included recurrent aphthous stomatitis, atrophic glossitis, geographic tongue, glossodynia, and xerostomia. The authors concluded that all these findings could enhance the management of oral diseases through targeted CeD therapy and places healthcare professionals at the vanguard of patients’ management, particularly when dealing with cases where diagnosis is delayed. In fact, the European Society for Paediatric Gastroenterology Hepatology and Nutrition (ESPGHAN) recommends considering testing for CeD in children and adolescents with recurrent aphthous stomatitis and dental enamel defects [12].

### 3.1. Recurrent Aphthous Stomatitis

Recurrent aphthous stomatitis (RAS) is one of the most prevalent oral pathological conditions, affecting 10–20% of the general population; it is more prevalent in individuals with nutritional deficiencies, malabsorption, and CeD [5]. RAS is characterized by multiple recurrent small, round, or ovoid ulcers with circumscribed margins, erythematous haloes, and yellow or gray floors typically. RAS usually occurs in the non-keratinized oral mucosa and affects feeding, speech, and swallowing and can cause considerable pain.

Investigations of children and adults with concomitant RAS and CeD and matched healthy controls did not indicate a common genetic background. Two Italian studies demonstrated that there was no correlation between the presence of CeD-specific HLA-DQB1*02 haplotypes and the occurrence of RAS [13,14]. The mechanisms, by which CeD leads to RAS, are not yet clear. It is not known whether RAS lesions are directly influenced by the gluten hypersensitivity itself, or if these are related to hematinic deficiency with low levels of serum iron, folic acid, and vitamin B_12_ or trace element deficiencies due to malabsorption in patients with CeD [15]. In a study from Spain, the examination of 20 patients with RAS and 10 healthy controls revealed that the salivary levels of tumor necrosis factor-α (TNF-α) were two to five times higher in the RAS group, indicating a possible implication of TNF-α in the RAS etiology [16]. TFA-α is an important factor of the inflammatory immune response in active CeD.

Polymorphonuclear neutrophils (PMN), present in oral smears, may also contribute to the formation of stomatitis, as demonstrated by an Argentinean study [17]. Oral ecosystem alterations in 25 children with CeD (4–12 years old) and 25 matched non-celiac controls were investigated for PMN. The CeD group showed significantly more PMN in smears (20% PMN per area) at diagnosis than the control group (0% PMN per area; *p* = 0.046). After a period of 18 months on a GFD, 90% of the CeD children, who showed PMN at diagnosis, did not present them anymore. However, further investigations are needed to better explore the mechanisms behind the relationship between RAS and CeD.

A systematic review and meta-analysis, including 30 scientific articles published until 2017, revealed that both pediatric and adult patients with CeD had a greater frequency of RAS (*p* < 0.00001 and <0.0002, respectively) compared with healthy controls [18]. Moreover, another recent systematic review reported that the average prevalence of RAS was 34% in the CeD population and 13% in the control group [7]. Table 1 summarized the most relevant studies published between 2010 and June 2023 about the prevalence of RAS compared with healthy individuals [10,11,19,20,21,22,23,24,25,26,27,28,29,30,31,32]. Most of them (twelve) concerned children and adolescents, while only four concerned other adults.

Whereas the higher prevalence of RAS in CeD patients has been demonstrated by a multitude of studies (Table 1), the prevalence of CeD in subjects with RAS has scarcely been investigated. An Iranian evaluation of CeD occurrence in RAS patients included a large group of children and adults with at least three RAS-specific attacks (n = 240) [33]. They were screened by CD-specific serology (IgA endomysium antibody, IgA transglutaminase antibody, and total IgA antibody levels) and duodenal biopsy. Seven patients (2.8%) had positive serology and all of them had duodenal biopsies compatible with CeD (age 13 to 40 years). Thus, the prevalence of CeD was much higher than that in the general population of Iran (0.9%). Four patients who adhered to a strict GFD showed noticeable improvement in their aphthous lesions over a period of six months.

A Turkish cohort of 82 adult patients (mean age 34.3 years) with RAS and 82 non-RAS controls (mean age 35.3 years) were screened for CeD-specific serum antibodies, and patients with positive serology underwent endoscopic duodenal biopsies [34]. One patient (1.2%) out of the RAS group was diagnosed with CeD, and none of the control subjects were diagnosed as having CeD. A recent review of medical records of 108 Turkish pediatric patients (aged 6 months to 18 years) suffering from RAS demonstrated that the frequency of biopsy-confirmed CeD was found to be 2.7% and thus approximately six times higher than the prevalence in healthy Turkish children (0.47%) [35].

All the studies cited here revealed a higher prevalence of RAS in both pediatric and adult CeD patients compared with controls (Table 1). RAS can, therefore, be considered as a “risk indicator” for CeD and should initiate screening for this disease even in the absence of gastrointestinal symptoms. In particular, early recognition of children with RAS and their referral to gastroenterologists might help in the early diagnosis of CeD. The effect of a GFD on the prevention or remission of RAS is still debated [36] and further comprehensive investigations should clarify the effect of a GFD.

### 3.2. Aphthous Ulcers

**Figure 2:**
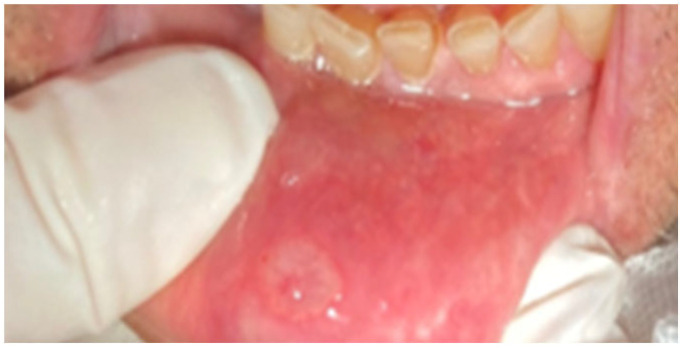
An aphthous ulcer in the oral cavity.

Aphthous ulcers (AUs) are a common variety of ulcers that form on the mucous membranes, typically occurring in the oral cavity. AUs appear solitary round or oval punched-out sores or ulcers inside the mouth on an area, where the mucosa is not tightly bound to the underlying bone such as the inside of the lips and cheeks or underneath the tongue (Figure 2).

They are benign, non-contagious, and can occur as single ulcers or in clusters. In most instances, AUs are recurrent with each episode, normally lasting for between 7 and 10 days. The cause of the condition is unclear, and there is no cure, but treatment options are available for the pain the AUs can cause. Several studies have shown clear associations between CeD and AUs. There are two possible pathophysiological mechanisms: firstly, the malabsorption and nutrients’ deficiencies caused by CeD, associated with low serum iron, folic acid, and vitamin B12; secondly, the possible involvement of immune-genetic factors [37].

Several previous studies compared the prevalence of AUs in patients with CeD and healthy controls. A study from Greece included 45 children and adolescents with CeD (mean age 10.3 years) and 45 matched healthy children [37]. The outcomes revealed a significant difference in the frequency of AUs in CeD patients (40%) compared with controls (4.4%; *p* = 0.001). A case–control study from Portugal included 80 patients with CD and 80 healthy subjects as controls, all aged between 6 and 18 years [38]. Data were gathered through a clinical record (questionnaire) and an intraoral observational examination. The prevalence of AUs was higher in the CD group (56%) compared with the control group (20%; *p* < 0.001). One third of the patients with CD and AUs referred that the beginning of the GFD improved this oral mucosa alteration. Two cohorts from the United States, consisting of 67 CeD patients (mean age 34.8 years) and 69 controls (mean age 28.1 years), were recruited from a private dental practice and from CeD support meetings [39]. AUs were observed in 42% of CeD patients and in 23% of controls (*p* = 0.02). A questionnaire about the history of AUs was used to examine 40 Brazilian patients with CeD (median age 16.4 years) and 40 matched healthy individuals [40]. The results showed that the frequency of AUs was higher in the CeD group (38%) compared with the control group (20%), but it was not statistically different (*p* = 0.084). The evaluation of oral manifestations in Indian adults (aged between 20 and 37 years) included 38 subjects with newly diagnosed CeD, 82 subjects on a GFD for at least one year, and 40 controls [41]. Overall, more patients with CeD (37%), both treatment naïve (44%) and GFD treated (34%), reported AUs in comparison with controls (13%), indicating that the frequency of AUs was significantly higher in CeD patients (*p* < 0.004 and 0.001, respectively) compared with controls.

### 3.3. Geographic Tongue

Geographic tongue (GT) is a common complaint affecting 2–3% of the general population. It is a non-cancerous condition of the mucous membrane usually on the dorsal surface and lateral borders of the tongue. It is characterized by areas of smooth reddish patches and loss of lingual papillae that can migrate over time. The name comes from the map-like appearance of the tongue, resembling land masses and oceans shown on a map. GT is not painful and not serious and there is no curative treatment. Numerous etiological factors have been suggested including immunological and genetic predisposition or emotional stress, but the real cause is still unknown. Several studies investigated the prevalence of CeD in patients with GT and of GT in patients with CeD, respectively.

A cross-sectional study from Italy included 50 children with ascertained CeD, 21 subjects with potential CeD, and 54 controls [11]. The mean age of the group members was 7.5, 6.9, and 8.8 years, respectively. The presence of GT was detected in 10% of ascertained CeD patients, 19% of potential CeD subjects, and 3.7% of controls (*p* < 0.05). A case–control study from Portugal included 80 patients with CD and 80 healthy subjects as controls, all aged between 6 and 18 years [38]. Data were gathered through a clinical record (questionnaire) and an intraoral observational examination. The prevalence of GT was higher in the CD group (7.5%) compared with the control group (1.3%), although it did not reach the statistical significance (*p* = 0.117). The prevalence of GT in Greece patients with CeD and controls was investigated by Zoumpoulakis et al. [37]. Overall, 45 children and adolescents with CeD, aged between 2 and 18 years, and 45 matched healthy controls were examined. The results revealed that three CeD patients (6.7%) and no control subject (0%) had GT. A recent Italian study included 114 pediatric individuals (aged between 6 and 14 years) who were divided into three groups with 38 participants each: CeD patients (CeD group), patients with malabsorption without CeD (non-CeD group), and healthy controls [31]. GT was more frequent in the CeD group (18%) and the non-CeD group (29%) compared with the control group (8%). In contrast to the four studies described above, none of the twenty Danish patients with CeD (mean age 49.2 years) showed GT, whereas among 20 matched healthy controls one subject (5%) had GT [30]. However, more CeD patients (20%) than controls (10%) presented a fissured tongue.

The prevalence of CeD in subjects with GT (age >18 years) was investigated in a study from Croatia [42]. CeD-specific serum antibodies against tissue transglutaminase (TGA) and gliadin (AGA) and human leukocyte antigen (HLA) typing were assessed for 60 GT patients and 60 healthy control subjects. The duodenal biopsy was performed in patients with positive serology. Altogether, nine GT patients (15%) suffered from CeD confirmed by serology and duodenal histology. There were statistically significant differences between the GT patients and controls for IgA TGA (15% vs. 0%; *p* = 0.03), IgG TGA (25% vs. 15%; *p* = 0.04), IgA AGA (22% vs. 5%; *p* = 0.04), and IgG AGA (23% vs. 3%; *p* = 0.02). There was no significant difference in HLA typing.

### 3.4. Atrophic Glossitis, Burning Tongue, and Glossodynia

Atrophic glossitis is characterized by a smooth glossy tongue and the partial or complete absence of lingual papillae on the dorsal surface of the tongue. It may be associated with burning sensation, pain (glossodynia), and/or erythema. Several conditions can cause atrophic glossitis such as malnutrition or malabsorption, which creates iron and B vitamin deficiencies, although other mechanisms may also be involved. Atrophic glossitis can be the only clinical sign for CeD [43].

Bramanti et al. examined the prevalence of atrophic glossitis in Italian children, 50 subjects with ascertained CeD (group A), 21 subjects with potential CeD (group B), and 54 controls (group C) [11]. The mean age of the group members was 7.5, 6.9, and 8.8 years, respectively. The prevalence of atrophic glossitis was 14% (A), 24% (B), and 1.9% (C) and that of burning tongue 14% (A), 9.5% (B), and 5.6% (C), respectively. Thus, the prevalence of both oral complaints was significantly higher in CeD patients compared with controls. A study from Portugal included 80 patients with CD and 80 healthy subjects as controls, all aged between 6 and 18 years [25]. Data were gathered through a clinical record (questionnaire) and an intraoral observational examination. The prevalence of atrophic glossitis was higher in the CD group (6.3%) compared with the control group (0%) but did not reach statistical significance (*p* = 0.059). In a recent Italian study, 114 pediatric patients (aged between 6 and 14 years) were divided into three groups with 38 participants each: CeD patients (CeD group), patients with malabsorption without CeD (non-CeD group), and healthy controls [31]. Atrophic glossitis and glossodynia were more frequent in the CeD group (21% and 16%) compared with the non-CeD group (13% and 11%) and the control group (2.6 and 0.0%).

In contrast, an Italian study on 300 CeD children and 300 non-CeD children did not reveal significant differences in the prevalence of atrophic glossitis (3.3 vs. 4.0%, *p* = 0.664) [33]. This finding was confirmed by a study from Greece on 45 children and adolescents with CeD, aged between 2 and 18 years, and 45 matched healthy controls [37]. None of both CeD patients and controls suffered from atrophic glossitis.

A cohort of 740 Dutch patients with CeD (median age 55 years) and 270 comparison participants (median age 53 years) were evaluated for the presence of glossodynia by means of an online questionnaire based on the Oral Health Impact Profile 14 [10]. The results revealed that 11% of CeD patients and 5% of controls suffered from glossodynia, which was significantly different (*p* < 0.01). An Iranian investigation, including 65 children with CeD (aged between 3 and 16 years) and 60 matched healthy controls, showed that the percentage of children with burning tongue was higher among the CeD group (12%) compared with the control group (3%); however, this did not reach the level of statistical relevance (*p* = 0.06) [27].

### 3.5. Angular Cheilitis

Angular cheilitis (AC) is an extra-oral condition that causes red, swollen patches in the corners of the mouth where lips meet and make an angle. It can occur on one side of the mouth or on both sides at the same time. Often the corners are red with skin breakdown and crusting and can also be itchy or painful, and complaints can last for days to years. AC can be caused by infection, irritation, or allergies among other causes.

Bramanti et al. [11] compared the prevalence of AC in Italian children with CeD and non-CeD, including 50 subjects with ascertained CeD (group A), 21 subjects with potential CeD (group B), and 54 controls (group C) (mean age 7.5, 6.9, and 8.8 years, respectively). The prevalence of AC was 6% in group A, 9.5% in group B, and 3.7% in group C. In another Italian study, 114 pediatric patients (aged between 6 and 14 years) were divided into three groups with 38 participants each: CeD patients (CeD group), patients with malabsorption without CeD (non-CeD group), and healthy controls [31]. AC was more frequent in the CeD group (10.5%) compared with the non-CeD group (7.9%) and the control group (0.0%). A study from Portugal included 80 patients with CD (mean age 13.3 years) and 80 healthy subjects as controls (mean age 12.5 years) [25]. Data were gathered through a clinical record (questionnaire) and an intraoral observational examination. The prevalence of AC was higher in the CD group (6.3%) compared with the control group (0%) without reaching statistical relevance (*p* = 0.059). The evaluation of Indian adults (aged between 20 and 37 years) included 38 subjects with newly diagnosed CeD, 82 subjects on a GFD for at least one year, and 40 controls [41]. Patients with CeD (14%), both treatment naïve (17%) and GFD treated (12%), and none of the controls reported AC.

### 3.6. Xerostomia

Xerostomia is the subjective sensation of dry mouth, which is often (but not always) associated with a change in the composition of saliva or reduced salivary flow. The prevalence of xerostomia in CeD patients in comparison with healthy controls was evaluated by several studies. Van Gils et al. investigated the occurrence of xerostomia in adult individuals from the Netherlands, including 740 patients with CeD (median age 55 years) and 270 comparison participants (median age 53 years) [10]. They also administered the Xerostomia Inventory (XI) questionnaire, an internationally validated questionnaire to examine dry mouth symptoms. The mean XI score was significantly higher in the CeD group (22.2) compared with the control group (17.2) (*p* < 0.001). The XI score was not associated with age at CeD diagnosis or with time on a GFD.

The examination of 20 Danish patients with CeD (mean age 49.2 years) and 20 matched healthy controls revealed that xerostomia was one of the most prevalent oral symptoms reported by the CeD patients (60%), and 20% also reported xerostomia-related difficulties in swallowing dry food substances and/or difficulties in speech [30]. All in all, 12 CeD patients (65%) and none of the healthy controls (0%) indicated to have been affected with xerostomia (*p* = 0.006). Two Turkish cohorts, consisting of 81 children with CeD (mean age 8.7 years) and 20 matched healthy children, were examined for xerostomia frequency by Ertekin et al. [31]. The results revealed that xerostomia occurred in 47 CeD patients (58%) and in 5 control subjects (25%). The difference was highly significant (*p* = 0.008).

The investigation by Shahraki et al. [27], including 65 Iranian children with CeD (aged between 3 and 16 years) and 60 matched healthy controls, showed that the percentage of children with xerostomia was higher among the CeD group (15%) compared with the control group (5%; *p* = 0.05). Ajdani et al. [44] analyzed 39 adult CeD patients on a GFD (mean age 39.2 years) and 39 healthy controls (mean age 34.3 years) using the Xerostomia Inventory questionnaire. They found that the obtained scores of xerostomia were significantly higher in CeD patients (mean 2.44) than in control subjects (1.91; *p* = 0.001).

Another study on 118 Indian patients with CeD (38 subjects with newly diagnosed CeD and 82 subjects on a GFD for at least one year) and 40 controls (median age of all participants 26 years) revealed a significantly different prevalence of self-reported xerostomia [41]. Overall, 81% of newly diagnosed patients and 63% of patients on a GFD reported dry mouth sensation in comparison with 7.5% of controls (*p* < 0.001). A Brazilian study, including 40 patients with CeD (median age 16.4 years) and 40 matched healthy individuals, evaluated subjective xerostomia through the question “does your mouth usually feel dry?” [40]. The results revealed that 13 out of 40 CeD patients (33%) suffered from dry mouth sensation compared with 2 out of 40 controls (5%) (*p* = 0.002). All in all, CeD patients reported dry mouth 9.15 times more than controls.

Associations between xerostomia and CeD without or with hematological problems were investigated in an Italian study [45]. Out of 76 adult CeD patients without hematological problems, 5 (6.6%) had xerostomia, while among 161 adult patients with hematological problems, 34 (21%) subjects had xerostomia. Thus, CeD patients with hematological problems had an almost four times risk of presenting xerostomia.

Altogether, xerostomia is a common complaint in both pediatric and adult patients with CeD, and its prevalence is significantly higher compared with non-celiac controls. A GFD apparently does not protect CeD patients from xerostomia.

### 3.7. Epithelial Disruption

To investigate whether the epithelial integrity of the oral mucosa is compromised in Spanish CeD patients, Sanchez-Solares et al. recruited six de novo diagnosed CeD patients (mean age 44.7 years), seven CeD patients under GFD for at least 1 year (mean age 48.6 years), and eight non-celiac subjects (mean age 34.5 years) [46]. Two biopsies of the cheek lining were taken from each subject for mRNA analysis and immune-histochemical characterization. The authors showed a significant difference in the expression of epithelial junction proteins; in particular, there was a significant decrease in E-cadherin expression in both groups of CeD patients (de novo: −20.3%, *p* < 0.01; GFD: −20.6%, *p* < 0.01) compared with non-celiac controls. Moreover, claudin-1 was also significantly decreased in both groups of CeD patients (de novo: −13.0%, *p* < 0.01; GFD: −11.8%, *p* < 0.01). No significant difference was found for occludin. Altogether, the results indicated that oral mucosa barrier integrity was impaired in all CeD patients. FoxP3+ population was greatly increased in CeD patients, suggesting that Treg lymphocytes were recruited to the damaged mucosa, even after avoidance of gluten, and might display a “repair” phenotype. In conclusion, the oral epithelial barrier of CeD patients is compromised, even when they adhere to a GFD. Further investigations with a larger number of patients and controls are needed to confirm these interesting findings.

### 3.8. Salivary Abnormalities

It is well known that saliva plays a crucial role in the maintenance of oral health. In recent years, numerous investigations on specific salivary properties of CeD patients compared with healthy controls have been published. The examination of unstimulated or stimulated salivary flow rate was the most used method for the investigation of salivary abnormalities. An unstimulated whole saliva flow rate of 0.3–0.4 mL/min is normal and below 0.1 mL/min is significantly abnormal. A chewing-stimulated saliva flow rate less than 0.5 mL/5 min or less than 1 mL/10 min is considered abnormal. Moreover, the examinations of salivary pH value and salivary buffering capacity were applied.

Several studies on children and adolescents identified significant differences between CeD patients and controls. Acar et al. [20] examined stimulated flow rate, buffer capacity, pH value, and cariogenic microflora of saliva in 35 CeD patients, aged 6–19 years, and 35 healthy individuals of the same age range. In the CeD group, a lower number of children (34%) were found to have a normal stimulated salivary flow rate compared with the healthy group (54%). Regarding buffering capacity, the difference between both groups was not statistically significant (*p* = 0.494). The saliva pH value also was in a similar range (mean 7.5 vs. 7.3). The prevalence of salivary *Streptococci mutans* (14% vs. 48%) and lactobacilli (34% vs. 51%) colonization was statistically lower (*p* = 0.012 and *p* = 0.010, respectively) in the CeD group.

The oral evaluation of 35 children with CeD and 35 healthy children, aged between 5 and 15 years, revealed that salivary flow rate and buffering capacity were lower in CeD patients compared with controls (*p* < 0.05) [24]. In another Turkish study, including 30 children with CeD and 30 without CeD (aged 6–16 years), the mean score of salivary flow rate was significantly lower in the CeD group (3.65) compared with the control group (7.46; *p* < 0.001) [47]. The saliva pH value also differed significantly (7.99 vs. 7.34; *p* < 0.001), whereas the mean score of buffering capacity was similar (5.99 vs. 5.87; *p* = 0.228). A recent clinical study, including 62 pediatric patients with CeD (mean age 12.3 years) and 64 non-celiac controls (mean age 12.1 years) showed a very low saliva flow (<0.7 mL/min) in 47% of subjects with CeD, whereas none of the controls had a lowered flow (*p* < 0.001) [32]. In contrast to the study of Acar et al. [20], the prevalence of *Streptococci mutans* >10^5^ was significantly higher in the CeD group than in the control group (73% vs. 47%; *p* = 0.003).

To evaluate salivary properties in a Brazilian cohort, 52 children with CeD, aged 2–15 years, and 52 matched controls were examined [23]. There was a highly significant difference in the salivary flow between the two groups; a low salivary flow was observed in 36% of the children with CeD and 12% of the controls (*p* = 0.006). The salivary pH mean value of the children with CeD (7.10) was significantly decreased compared with the control group (7.26) (*p* = 0.165). The buffering capacity of both groups did not show a significant difference (12% vs. 20%; *p* = 0.358).

On the contrary, other studies did not find any differences in salivary properties in CeD patients compared with controls. The examination of 25 Argentinian children with CeD (mean age 4–12 years) and 25 matched healthy controls revealed no statistically significant difference (*p* > 0.05) in salivary flow rate [17]. In a study from Israel, three groups of children were evaluated for salivary abnormalities: newly diagnosed CD (n = 30, mean age 6.9 years), CD treated with a GFD (n = 30, mean age 9.5 years), and healthy controls (n = 30, mean age 6.0 years) [43]. No significant difference was found in pH value and buffer capacity of saliva and counts of salivary *Strepococci mutans* and lactobacilli [48]. Two Brazilian cohorts, consisting of 40 patients with CeD (median age 16.4 years) and 40 matched healthy individuals, were recruited for the evaluation of unstimulated and stimulated whole salivary flow [27]. Patients with CeD had normal patterns of unstimulated and stimulated flow rates (mean 0.67 mL/min and 1.14 mL/min, respectively) and did not differ from the controls.

The examination of 20 Danish adult patients with CeD (mean age 49.2 years) and 20 matched healthy controls revealed that the CeD patients even had significantly higher unstimulated and stimulated saliva flow rates (0.47 vs. 0.26 mL/min and 0.35 vs. 0.26 mL/min, respectively) than the healthy controls (*p* = 0.01 and 0.05, respectively) [30]. The stimulated parotid saliva flow rate of the CeD patients was also higher (0.80 vs. 0.55 mL/min), although not statistically significant (*p* = 0.06). The salivary count of *Streptococcus mutans*, lactobacilli, and *Candida blastospores* and *hyphae* did not differ significantly between CeD patients and healthy controls. Furthermore, the authors evaluated the presence of inflammatory and structural changes in minor salivary glands. The immunohistochemical analysis of the glands revealed more extensive inflammation and more focal lymphocytic infiltration in the labial salivary glands of CeD patients than those of healthy individuals.

An international study investigated the salivary enzymatic activities and oral microbial profiles in adult healthy subjects versus adult patients with classical and refractory CeD [49]. Stimulated whole saliva was collected from patients with CD in remission (n = 21) and refractory CeD (n = 8) and was compared with healthy controls (n = 20). Salivary glutenase activities were higher in CD patients compared with controls, both before and after normalization for protein concentration or bacterial load. The oral microbiomes of CD and refractory CD patients showed significant differences from that of healthy subjects; for example, they found higher salivary levels of lactobacilli (*p* < 0.05), which may partly explain the observed higher gluten-degrading activities. The presented data suggested that oral microbe-derived enzyme activities were elevated in subjects with CeD, which may impact gluten processing and the presentation of immunogenic gluten epitopes to the immune system in the small intestine.

Ajdani et al. compared the levels of minerals and proteins in the saliva of 39 Iranian CeD patients on a GFD (mean age 39.2 years) and 39 healthy controls (mean age 34.2 years) [44]. The mean concentrations of total protein, albumin, calcium, phosphorus, sodium, and potassium of CeD patients and controls were not statistically different. The mean level of amylase was significantly lower (*p* < 0.001) and that of interleukin 6, 18, and 21 significantly higher (*p* = 0.001) in the CeD group compared with the control group. The mean salivary level of IgA transglutaminase antibodies (TGA) was significantly higher in CeD patients than in controls (*p* = 0.001). The authors proposed that the measurement of salivary IgA TGA might be a non-invasive, inexpensive alternative screening method for CeD diagnosis.

In conclusion, reports on associations between salivary flow and CeD are controversial, and recent studies are necessary to generate clearness. Moreover, further investigations are needed to clarify the role of cariogenic salivary microflora. Other salivary parameters such as a salivary pH value and buffering capacities appear to be of no relevance. Future attention should be paid to salivary levels of inflammatory cytokines and TGA found in CeD patients and inflammatory and structural changes in minor salivary glands.

## 4. Sensorineural Hearing Loss

Sensorineural hearing loss (SNHL) is a specific type of hearing loss that results from damage to the hair cells within the inner ear or to the vestibulocochlear nerve. SNHL accounts for approximately 90% of reported hearing loss in the general population, is usually permanent, and can be mild, moderate, severe, profound, or total. The pathological mechanism behind SNHL is not entirely clear and several mechanisms such as malabsorption and immune-mediated neurological damage, comprising autoantibodies, autoreactive T cells, and immune complex deposition, have been proposed [50,51]. Most used methods for the examination of hearing complaints were pure-tone audiometry (PTA), tympanometry, transiently evoked otoacoustic emission (TEOAE), distortion product otoacoustic emission (DPOAE), and contralateral suppression of the TEOAE.

A possible correlation between SNHL and CeD investigated in adults was first reported in an Italian study from 2007 [52]. Subsequent investigations on associations between CeD and SNHL, including one study on adult patients and eight studies on children and adolescents, were summarized in a systematic review presented by De Luca et al. [51]. Another study suggest that hearing screenings should be recommended for children with CD in order to prevent the potentially unfavorable effects of hearing loss on the emotional, behavioral, cognitive, and sensorimotor development of these patients [53]. The presented outcomes were partly contradictory, and an unambiguous close relation between both disorders could not be deduced. Table 2 reports the studies evaluating the prevalence of sensorineural hearing loss in CeD patients compared with controls.

Knowledge about SNHL, being an extra-intestinal manifestation of CeD, remains insufficient. Some examinations revealed that CeD seems to have an important impact on the auditory system; however, others demonstrated that the hearing level of CeD patients does not seem to differ from that of non-celiac controls. Based on the still unclear relations between SNHL and CeD, hearing function tests should not be routinely performed in the clinical setting of CeD but only in CeD patients with clinical signs of hearing deficiency. The effect of a GFD on hearing loss in CeD patients is scarcely investigated. The few presented studies did not indicate clear differences between treated and untreated patients. Altogether, further rigorous international studies are needed to achieve statistically robust evidence of hearing loss prevalence in CeD patients compared with controls, the effect of a GFD, and the pathophysiology of SNHL in CeD patients.

## 5. Nasal Abnormalities

Nasal mucociliary clearance (NMC) is a key first-line defense of the respiratory tract that clears the nasal epithelium and protects the respiratory system from damage by inhaled substances. This defense mechanism is dependent on appropriate mucus production and coordinated ciliary activity, which enables the transport of the overlying debris-laden mucus to the oropharynx, where it is swallowed or expectorated. Impairment of NMC can play a role in the development of airway infections frequently found in patients with CeD. One recent study on relations between NMC and CeD was published by Comba and Atan [58]. A total of 43 Turkish patients with CeD and 22 healthy controls (average age 11.8 ± 4 years) were examined using the saccharin test (taste time in seconds). Clearance time of patients with CeD (531 ± 155 s) was significantly prolonged in comparison with that of healthy children (448 ± 80 s) (*p* = 0.006). No relationships were found of diagnosis age, CeD type, histopathological phase, and compliance with the GFD with clearance time of patients with CeD. Hemorrhagic events are common as part of the clinical spectrum of adult patients with CeD. In few cases, CeD has been linked to nasal septal perforation and epistaxis [50]. Case reports of 46 Romanian CeD patients with hemorrhagic manifestations, aged between 19 and 74 years, revealed that one patient suffered from bleeding nasal septal perforation and further three cases from life-threatened epistaxis, related to vitamin K and vitamin D malabsorption [59]. There was good evidence that these complaints improved with a GFD.

## 6. Obstructive Sleep Apnea

Obstructive sleep apnea (OSA) is one of the most common sleep-related breathing disorders. It is characterized by repetitive stop and start breathing during sleep, which may result in snoring, sleep disruption, and observable apneic episodes. To determine whether children with CeD are at risk for sleep-disordered breathing, 19 Italian children with newly diagnosed CeD (mean age 9.8 years) were recruited by Parisi et al. (2015) [60]. Each patient underwent a standardized general and neurological examination and a validated questionnaire about OSA (scores <0 predict normality, scores >0 predict OSA). At CeD diagnosis, six patients (31.6%) showed a positive OSA score and thus the prevalence was much higher compared with the general pediatric population (1.2–5.7%). After six months of a strict GFD, all the children showed complete resolution of OSA. A subsequent investigation by Yerushalmy-Feler et al. examined 39 Israeli children with newly diagnosed CeD (mean age 6.6 years) and 24 non-celiac controls (mean age 7.3 years) by means of the sleep-related breathing disorder scale of the pediatric sleep questionnaire (PSQ) [56]. The rate of positive PSQ scores was higher in the control group (33.3%) compared with the celiac group (11.8%), which contrasted with the previous study by Parisi et al. Different tools for the assessment of disordered sleep and divergent numbers of participants may be causative for the differences. Six months after initiating a GFD, no patients in the CeD group had a positive PSQ score. This finding agreed with that of Parisi et al., who also showed resolution of sleep-disordered breathing with a GFD. A potential explanation for the GFD effect may be improvement in the lymphatic hyperplasia that is associated with CeD and that contributes to OSA in these patients [61]. All in all, further studies are necessary that can add to our limited knowledge on the relations between CeD and OSA.

## 7. Esophageal Disorders

Several esophageal disorders such as GERD, Barrett’s esophagus, and eosinophilic esophagitis (EoE) have been described in CeD patients. Esophageal symptoms may include heartburn, acid regurgitation, but also chest pain and dysphagia for solids and/or liquids. Diagnostic tests for esophageal disorders include upper endoscopy with biopsies to look for abnormalities, esophageal manometry, and 24-hour esophageal pH monitoring.

Up until 30 years ago, case reports and cohort studies have suggested an association between CeD and esophageal abnormalities. Ludvigsson et al. conducted a large endoscopic study on 1000 Swedish adults randomly selected from the general population [57]. Among the participants, 40% had GERD, 16% had erosive esophagitis, 4.8% had esophageal eosinophilia (defined as any eosinophilic infiltration of the esophageal epithelium), 1.6% had Barrett’s esophagus, and 1.1% had eosinophilic esophagitis (defined by the presence of at least 15 eosinophils/high power field in biopsies from the distal esophagus). The frequency of CeD diagnosis was not significantly different in GERD patients vs. controls (*p* = 0.81); in patients with erosive esophagitis vs. controls (*p* = 0.75); in individuals with esophageal eosinophilia vs. controls (*p* = 0.21). No cases of CeD were found in patients with Barrett’s esophagus and eosinophilic esophagitis.

### 7.1. Gastro-Esophageal Reflux Disease

GERD is one of the most common chronic disorders found in specialist and general medicine clinics. The prevalence of GERD varies from 5% to 20% across the globe [62]. According to the Montreal Consensus definition, GERD develops when reflux of gastric contents causes troublesome symptoms and/or complications [63]. Heartburn, defined as the burning sensation felt in the retrosternal area with a tendency to radiate towards the throat, and regurgitation have been recognized as the most specific symptoms of GERD.

GERD symptoms are common also in CeD patients, with 30% of them considering symptoms to be moderate to severe [64,65]. The mechanisms underlying reflux symptoms in CeD patients are unknown, but esophageal motor disturbances and a delayed gastric emptying have been suggested. Light microscopy and electronic microscopy on endoscopic biopsies from the distal esophagus demonstrated that the intercellular space was altered, and the expression of tight junction proteins was lowered in patients with active CeD compared with controls [66]. Accordingly, the authors concluded that the loss of mucosal integrity may contribute to reflux symptom generation.

Nachman et al. assessed the prevalence of GERD symptoms in an Argentinian cohort of 133 adult patients with CeD (mean age 38.1 years) and 70 healthy controls (mean age 39.5 years) using a sub-dimension of the Gastrointestinal Symptoms Rating Scale for heartburn and regurgitation domains [65]. At CeD diagnosis, 30.1% of patients had moderate to severe GERD (score >3) compared with 5.7% of controls (*p* < 0.01). A rapid improvement was evident at three months after initial treatment with a GFD (*p* < 0.0001) with reflux scores comparable to healthy controls. Mooney et al. showed that the prevalence of undiagnosed CD among GERD patients was similar to that in the general population; therefore, the authors did not recommend to routinely perform duodenal biopsies of GERD patients [62]. In patients with newly diagnosed CeD, they found a high prevalence of esophageal dysmotility; in fact, 9% had a hypotensive lower esophageal sphincter and 40.6% had esophageal motor abnormalities, with 25% significantly hypocontractile.

Endoscopic and histologic findings in Canadian pediatric patients (mean age 9.1 years), undergoing esophageal endoscopy for the first-time diagnosis of CeD, were published by Boschee et al. [67]. Six/seventy-six subjects (7.9%) presented reflux esophagitis at endoscopy; the histological evaluation of esophageal biopsies revealed reflux esophagitis in eight/seventy-six subjects (10.5%).

### 7.2. Eosinophilic Esophagitis

**Figure 3:**
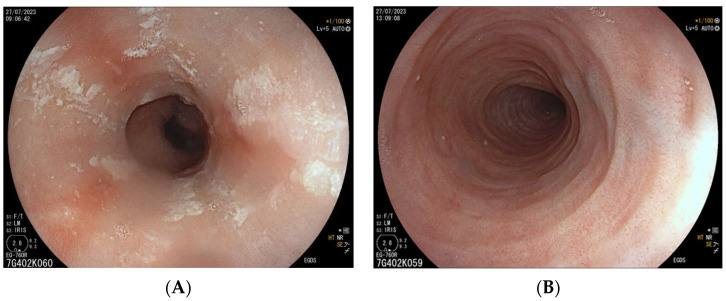
Some of the endoscopic findings of EoE: longitudinal furrows, white exudates (**A**); esophageal rings (**B**).

Eosinophilic esophagitis (EoE) is a rare inflammatory condition of the esophagus clinically characterized by chronic symptoms of esophageal dysfunction and histologically by ≥15 eosinophils per high power field (eos/hpf) at esophageal biopsies. First described in its current form in the early 1990s, the incidence and prevalence of EoE have increased over the decades. Currently, the incidence range has been estimated at 5 to 10 cases per 100,000 and prevalence range at 0.5 to 1 case per 1000 [64]. Due to eosinophil infiltration, the esophagus does not contract properly, becomes narrowed, and develops rings or stenosis. Typical signs and symptoms are dysphagia, vomiting, regurgitation, nausea, epigastric pain, and reflux. The common endoscopic, although not pathognomonic, are longitudinal furrows, white exudates (plaques), rings (trachealization), strictures, edema (mucosal pallor or decreased vascularity), and a narrow-caliber esophageal lumen (Figure 3).

The association between EoE and CeD has been the focus of numerous studies. In particular, the prevalence of EoE in CeD patients and vice versa has been used as an indicator for a potential relationship. An update on the association between EoE and CeD has been presented by Pellicano et al. [68]. The search identified 30 publications on this subject. Most of the studies on pediatric populations revealed that the prevalence of EoE in subjects with CeD was approximately 10 times that of the general population.

Thompson et al. [69] reviewed a database of patients with CeD to determine the number of patients with comorbid diagnoses of EoE. Altogether, 297 children and adolescents (aged 0–19 years) and 1142 adults (aged >19 years) with biopsy-confirmed CeD underwent esophageal biopsy. Among children and adolescents, 10 subjects (0.9%) and, among adults, 14 subjects (1.0%) had EoE. Age-adjusted and sex-adjusted standardized incidence ratios (SIR) were calculated in comparison with published US population-derived incidence data. An SIR greater than 1 indicated a higher than expected number of cases within the reference population as compared with the general population. EoE was diagnosed in 4 out of 297 children (1.3%) and 10 out of 1142 adults (0.9%), resulting in 1.0% of all participants. EoE was more common compared with the general population: SIR for children was 35.6 and for adults 13.1; overall, SIR was 16.0. Apart from Thompson’s work, 11 studies on the frequency of EoE in patients with CeD were published in the period between 2009 and June 2023 (Table 3). Most of them (seven) concerned children and adolescents, the other adults (one), and mixed populations (three).

As shown in Table 3, a large extent of variability was found in the prevalence of EoE (0.0–10.7%) in patients with CeD. The majority of authors indicated that the prevalence of EoE in CeD patients was higher compared with the general population; however, adequate control subjects were widely missing. In any case, coexistent EoE should be considered in patients with CeD who have persistent esophageal symptoms. Some studies suggested a common underlying mechanism of CeD and EoE, whereas others maintained that their co-existence is coincident [79]. Altogether, further studies are needed to investigate the relationship between EoE and CeD, in particular the mechanism(s) linking these disorders.

The prevalence of CeD in patients with EoE was evaluated by several studies that are reported in Table 4. Altogether, the six reviewed studies revealed a wide variability of CeD prevalence (0–13.6%) in patients with EoE. Although an adequate control cohort is often missing, four studies indicated that the prevalence of CeD in EoE patients was higher than in the general population.

## 8. Conclusions

Recent studies suggested that ORL and esophageal manifestations in both children and adults may be part of the manifold spectrum of extra-intestinal symptoms of CeD. Regarding the oral soft tissue, the increased prevalence of RAS, aphthous ulcers, geographic tongue, angular cheilitis, and xerostomia in CeD patients compared with healthy controls indicate a clear association between CeD and these oral complaints. They can be classified as risk factor signals for CeD, and CeD screening tests for patients with these disorders should be selectively considered even in the absence of gastrointestinal symptoms. The relations between CeD and atrophic glossitis, glossodynia, and salivary abnormalities remain unclear, and further clinical investigations should be encouraged. Although the associations between CeD and sensorineural hearing loss have recently been investigated by an increased number of studies, clear conclusions could not be reached and, therefore, further comprehensive investigations are necessary. Nasal complaints such as reduced mucociliary clearance and epistaxis as well as obstructive sleep apnea may also be ORL manifestations in CeD, but studies are rare and further examinations are needed including sufficient CeD patients and controls. Regarding esophageal disorders, the association between CeD and GERD remains unclear and the role of a GFD in patients with both CeD and GERD is still vague. Further investigations with an enlarged number of patients and controls are necessary. Current evidence did not suggest an increased prevalence of CeD in GERD patients, so the CeD screening should not be routinely recommended in GERD patients. On the contrary, several studies indicate that the prevalence of EoE in CeD patients was higher compared with the general population, although an adequate control cohort is often missing. The pathophysiological link between EoE and CeD might be further investigated, since current evidence is still conflicting. However, a coexistent EoE should be considered in patients with CeD who have persistent esophageal symptoms.

How GFD may favor an improvement and non-adherence to GFD favor maintenance of ORL and esophageal conditions remains in part unclear. A number of studies indicated that there is no difference between the prevalence of ORL manifestations in treated and untreated patients and, consequently, they appear to be progressive and permanent. Other investigations generated contradictory results; however, the compliance of the patients with the GFD was not controlled in most cases. Future investigations are necessary, in which a strict GFD should be ensured. The pathogenesis behind the association between ORL manifestations and CeD is not yet entirely clarified, and several mechanisms such as malabsorption, immunological, genetic, and environmental factors have been proposed. Further studies are needed about the pathophysiological link between CeD and ORL manifestations.

Otorhinolaryngologists should be aware of the possibility of CeD in patients with ORL complaints, especially when other potential CeD-related symptoms are present, and consider referring these patients to a general practitioner or gastroenterologist for further evaluation. Early diagnosis of CeD may help prevent its progress and long-term complications and can trigger an effective change in the quality of life of CeD patients.

## Figures and Tables

**Table 1:** Prevalence [%] of recurrent aphthous stomatitis (RAS) in CeD patients vs. controls.

Author (Year)	Country ^a^	Pop. ^b^	n ^c^	% ^d^
Costacurta (2010) [19]	IT	C	300 vs. 300	8.3 vs. 3.0% s
Acar (2012) [20]	TR	C	35 vs. 35	37 vs. 11% s
Ertekin (2012) [21]	TR	C	81 vs. 20	48 vs. 5% s
Cantekin (2012) [22]	TR	C	25 vs. 25	44 vs. 0% s
Bramanti (2014) [11]	IT	C	50 ^e^ vs. 54	52 vs. 7% s
De Carvalho (2015) [23]	BR	C	52 vs. 52	40 vs. 17% s
Dane (2016) [24]	TR	C	35 vs. 35	31 vs. 0% s
Saraceno (2016) [25]	IT	C	83 vs. 83	69 vs. 43% s
Amato (2017) [26]	IT	A	49 ^f^ vs. 51	53 vs. 26% s
Van Gils (2017) [10]	NL	A	740 vs. 270	35 vs. 23% s
Shahraki (2019) [27]	IR	C	65 vs. 60	17 vs. 13% ns
Alsadat (2021) [28]	SA	C	104 vs. 104	42 vs. 15% s
Villemur Moreau (2021) [29]	FR	A	28 vs. 59	50 vs. 22% s
Liu (2022) [30]	DK	A	20 vs. 20	85 vs. 35% s
Ludovichetti (2022) [31]	IT	C	38 vs. 38	24 vs. 8% s
Elbek-Cubukcu (2023) [32]	TR	C	62 vs. 64	31 vs. 0% s

^a^ Country code ISO 3166-1. ^b^ C, children/adolescents; A, adults. ^c^ Number of subjects. ^d^ s, significant (*p* < 0.05); ns, non-significant (*p* > 0.05). ^e^ Ascertained CeD. ^f^ On a GFD.

**Table 2:** Prevalence [%] of sensorineural hearing loss in CeD patients vs. controls.

Author (Year)	Country ^a^	Pop. ^b^	n ^c^	% ^d^
Leggio (2007) [52]	IT	A	24 vs. 24	47 vs. 9% s
Volta (2009) [54]	IT	A	59 vs. 59	9 vs. 3% ns
Hizli (2011) [55]	TR	C	32 vs. 32	41 vs. 3% s
Karabulut (2011) [56]	TR	C	41 vs. 31	29 vs. 4% s ^e^
Yazici (2019) [57]	TR	A	103 vs. 79	4 vs. 0% ns

^a^ Country code ISO 3166-1. ^b^ C, children/adolescents; A, adults. ^c^ Number of subjects. ^d^ s, significant (*p* < 0.05); ns, non-significant (*p* <0.05). ^e^ PTA1 test.

**Table 3:** Prevalence [%] of EoE in CeD patients.

Author (Year)	Country ^a^	Pop. ^b^	n ^c^	%
Leslie (2010) [70]	AU	C	121	8.3
Abraham (2012) [71]	CA	C	215	4.1
Thompson (2012) [69]	US	M	1439	1.0
Stewart (2013) [72]	CA	M	763	0.4
Ludvigsson (2013) [73]	SE	A	18	0.0
Dharmaraj (2015) [74]	US	C	56	10.7
Jensen (2015) [75]	US	M	546	6.0
Boschee (2017) [67]	CA	C	76	10.5
Hommeida (2017) [76]	US	C	10,201	1.8
Patton (2019) [77]	US	C	350	6.3
Cristofori (2021) [78]	IT	C	313	0.3

^a^ Country code ISO 3166-1. ^b^ C, children/adolescents; A, adults; M, mixed. ^c^ Number of subjects.

**Table 4:** Prevalence [%] of CeD in EoE patients.

Author (year)	Country ^a^	Pop. ^b^	n ^c^	%
Abraham (2012) [71]	CA	C	95	10.5
Ludvigsson (2013) [73]	SE	A	11	0.0
Stewart (2013) [72]	CA	M	421	0.7
Jensen (2015) [75]	US	M	4101	1.8
Johnson (2016) [80]	US	A	44	13.6
Hommeida (2017) [76]	US	C	595	1.6

^a^ Country code ISO 3166-1. ^b^ C, children/adolescents; A, adults; M, mixed. ^c^ Number of subjects.

## Data Availability

Not applicable.
